# Physicochemical and bioactive properties of dehydrated mango pulp (*Mangifera indica*) using solar technology in the Amazonas Region, Peru

**DOI:** 10.3389/fnut.2026.1960440

**Published:** 2026-10-14

**Authors:** Merbelita Yalta Chappa, Homar Santillan Gomez, Sandra Mori-Vigo, Luz Quispe-Sanchez, Fernando Isaac Espinoza Canaza, Wildor Gosgot Angeles, Robin Oblitas-Delgado, Doris Tello Rodríguez, Manuel Oliva Cruz, Mariños López Mas, Carla Ordinola Ramírez, Miguel Ángel Barrena Gurbillón

**Affiliations:** Instituto de Investigación para el Desarrollo Sustentable de Ceja de Selva (INDES-CES), Universidad Nacional Toribio Rodríguez de Mendoza de Amazonas (UNTRM), Chachapoyas, Peru

**Keywords:** antioxidants, bioactive compounds, mango, Peruvian Amazon, physicochemical properties, solar drying

## Abstract

**Introduction:**

*Mangifera indica* L. is a highly perishable tropical fruit, and sustainable preservation strategies are required to reduce postharvest losses while maintaining product quality. This study evaluated the influence of three solar drying configurations on the physicochemical, textural, and bioactive properties of mango (*Mangifera indica* L. var. Keitt) under the humid tropical highland conditions of the Peruvian Amazon.

**Methods:**

The evaluated systems were a natural convection dryer without thermal storage (NCD), a forced convection dryer with limestone thermal storage in the solar collector (FCD-TSC), and a forced convection dryer with limestone thermal storage in the drying chamber (FCD-TSD). Mango slices (3 ± 0.5 mm) were dehydrated for up to 3120 min, and changes in pH, titratable acidity, total soluble solids (TSS), moisture, color, firmness, total phenolic content (TPC), and antioxidant capacity were evaluated.

**Results:**

Drying method, drying time, and their interaction significantly affected most properties. Moisture decreased from 86.81% in fresh mango to 21.67%, 18.70%, and 12.24% after 3120 min in NCD, FCD-TSC, and FCD-TSD, respectively, with FCD-TSD showing the greatest moisture removal. TSS and titratable acidity increased during dehydration, while firmness generally increased with drying time. FCD-TSD showed favorable lightness and the highest TPC at 3120 min (27.89 mg GAE/g), whereas the highest antioxidant capacity was observed in NCD (9.43 μmol TE/g).

**Discussion:**

The apparent increases in bioactive properties were mainly associated with moisture loss, concentration effects, and changes in extractability during drying. Overall, forced convection combined with thermal storage, particularly when the storage material was located in the drying chamber, improved moisture removal while influencing the physicochemical and functional properties of dehydrated mango.

## 1 Introduction

Mango (*Mangifera indica* L.) is one of the most important tropical fruits due to its nutritional value and high content of bioactive compounds, including phenolics, flavonoids, and carotenoids (Suh et al., 2022, Hussain et al., 2021). These compounds are associated with antioxidant, anti-inflammatory, and health promoting properties, increasing the relevance of mango in human nutrition. However, its high moisture content and rapid postharvest metabolism make it highly perishable, leading to significant losses during storage and commercialization (Kolle et al., 2018, Mirza et al., 2021). Therefore, effective preservation strategies are required to extend shelf life while maintaining product quality.

Among available technologies, dehydration is widely used to reduce moisture content, thereby limiting microbial growth and enzymatic activity responsible for deterioration. However, conventional drying methods often rely on fossil fuels, resulting in high energy consumption and environmental impact (Karami et al., 2025). In this context, solar drying has emerged as a sustainable and cost effective alternative for fruit preservation, particularly in regions with abundant solar radiation. This technology reduces dependence on non-renewable energy sources and contributes to environmentally friendly food processing systems (Singh et al., 2022). Depending on system configuration, solar dryers may operate under natural or forced convection and can incorporate thermal storage to improve temperature stability, drying efficiency, and final product quality (Verma et al., 2020). These technological variations directly influence heat and mass transfer processes, which in turn affect the physicochemical and bioactive characteristics of dehydrated products.

Despite these advances, limited information is available regarding the impact of solar drying on the physicochemical and bioactive properties of mango under humid tropical highland conditions such as those of the Amazonas region, Peru. This knowledge gap restricts the implementation of appropriate technologies for postharvest loss reduction and agro-industrial development.

We hypothesized that forced convection solar drying systems equipped with thermal storage would enhance dehydration efficiency and improve the preservation of physicochemical and bioactive properties compared with natural convection systems. The present study aimed to characterize the physicochemical and bioactive properties of mango dehydrated using solar technology, employing three solar drying systems: a natural convection air dryer (NCD), a forced convection dryer with thermal storage in the solar collector (FCD-TSC), and a forced convection dryer with thermal storage in the drying chamber (FCD-TSD).

## 2 Materials and methods

### 2.1 Study area

The solar dryers were installed on the campus of the Universidad Nacional Toribio Rodríguez de Mendoza (UNTRM) in the Amazonas region, district and province of Chachapoyas, specifically within the PROCICEA project area (Figure 1). The geographic coordinates were 6°14′05.13″ S latitude and 77°51′16.13″ W longitude. The site is characterized by an average relative humidity of 71%, minimum and maximum temperatures of 9.5 °C and 28.04 °C, respectively, an adjusted mean daily precipitation of 0.77 mm, and an average annual cloud cover of 77.5%.

**FIGURE 1:**
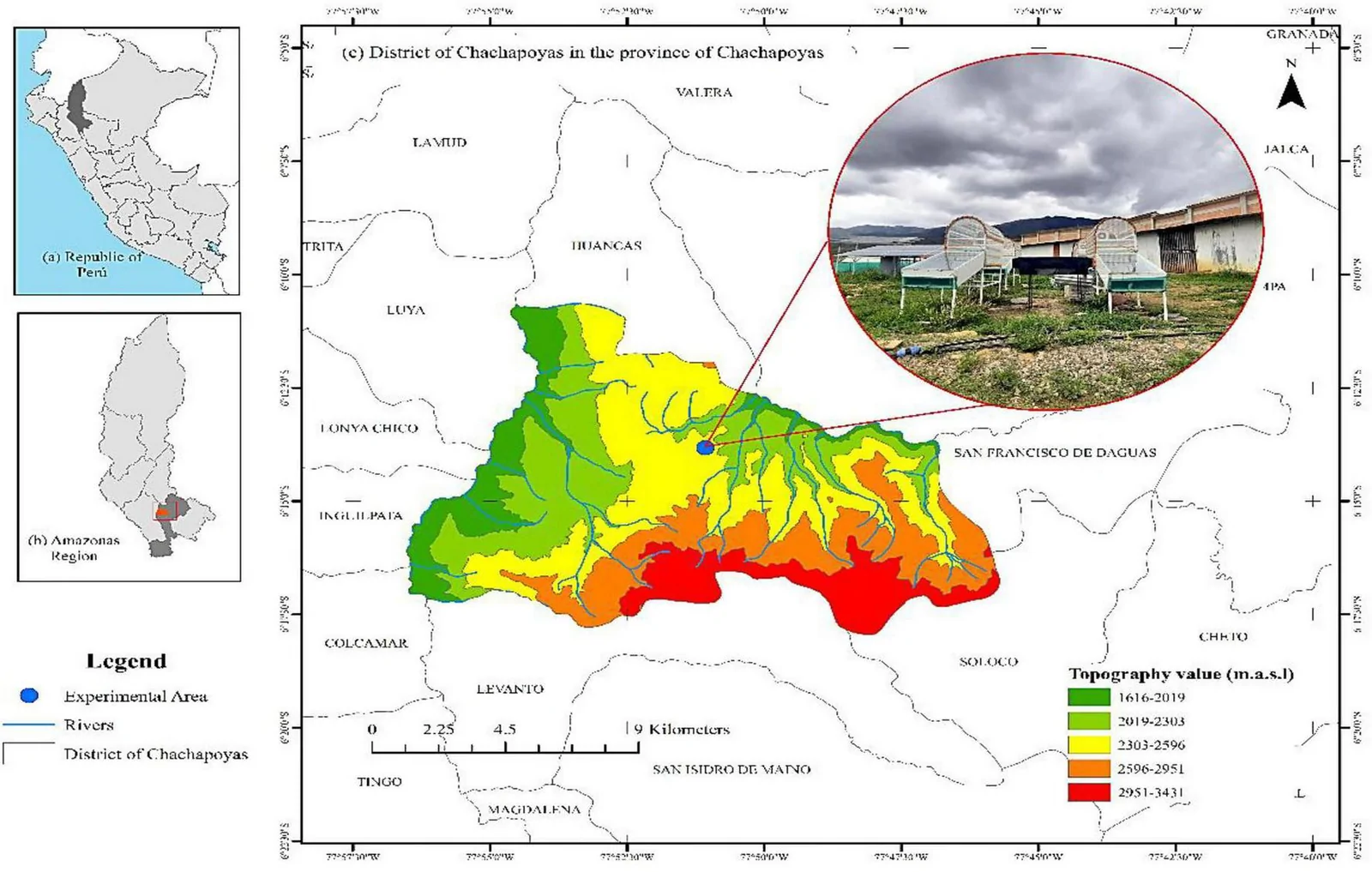
Geographic location of the experimental site and solar drying systems at the Universidad Nacional Toribio Rodríguez de Mendoza (UNTRM), Chachapoyas, Amazonas, Peru.

Despite the high cloudiness conditions characteristic of the study area, progressive moisture reduction was achieved during the experimental period in all evaluated drying systems.

### 2.2 Characteristics of the solar dryers

Prototype solar dryers were used, each consisting of a 1 m^2^ solar collector connected to a closed drying chamber made of translucent polycarbonate and equipped with three stainless steel mesh trays. Three drying configurations were evaluated, sharing the same basic structural design but differing in the airflow mechanism and the presence and location of limestone used as sensible thermal storage material. The limestone was placed in a separate compartment within the corresponding drying system and did not come into direct contact with the mango samples. Prior to installation, the limestone was cleaned to minimize the presence of surface particles, and the system configuration prevented the transfer of limestone dust into the drying chamber.

The first configuration corresponded to natural convection drying (NCD), which operated without mechanically induced airflow or thermal-storage material. Air circulation occurred passively through the connection between the solar collector and the drying chamber. The second configuration consisted of forced convection drying with thermal storage in the solar collector (FCD-TSC), in which limestone was placed within the solar collector. The third configuration corresponded to forced convection drying with thermal storage in the drying chamber (FCD-TSD), in which limestone was located inside a separate compartment of the drying chamber. In both forced-convection configurations, air circulation was enhanced by an air extractor installed in the upper section of the drying chamber, providing a mass airflow rate of 0.038 kg⋅s^_−_^1^^ to facilitate air exchange and moisture removal.

The drying chambers were mounted on metallic supports and covered with translucent polycarbonate to allow solar radiation transmission while protecting the mango samples from direct exposure to external environmental conditions. The solar collectors were positioned to maximize solar exposure under the local climatic conditions during the experimental period (Figure 2). Because the systems were solar-driven, the thermal conditions varied naturally throughout the day according to the prevailing environmental conditions rather than being maintained at a constant drying temperature. In the forced-convection systems, the air extractor was automatically activated when the solar collector temperature reached 55 °C. Thus, 55 °C represented the operational threshold for fan activation rather than a fixed drying temperature.

**FIGURE 2:**
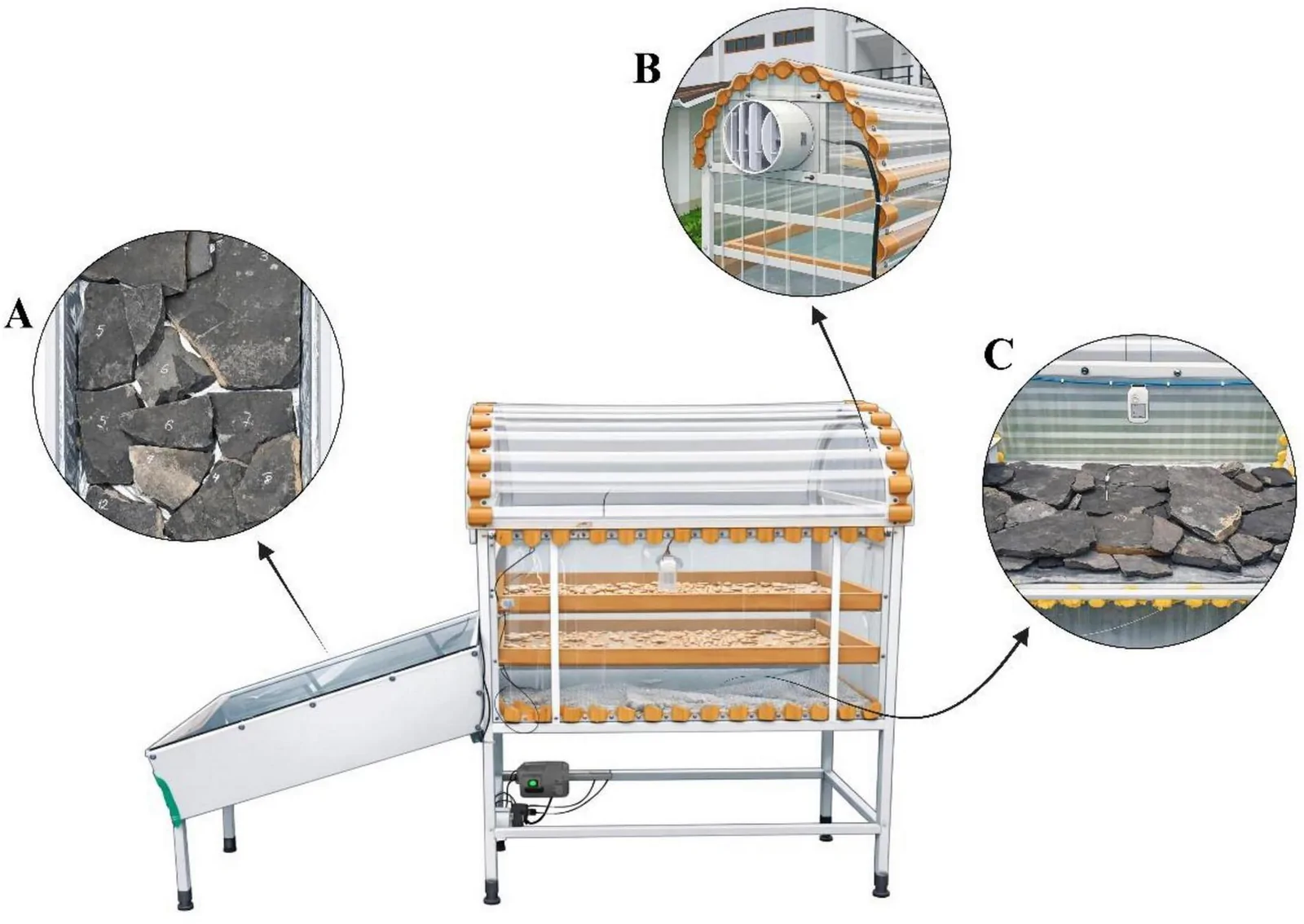
Solar dryer with collector: **(A)** limestone in the solar collector, **(B)** forced air extraction at the top of the drying chamber, and **(C)** limestone in the drying chamber.

The drying experiment was conducted for a maximum period of 3120 min (52 h), with measurements performed at 240, 480, 1440, 1680, 1920, 2880, and 3120 min. At the end of each daily drying period, the mango samples remained inside their respective dryers, which were kept closed overnight, and drying resumed the following day under the prevailing solar conditions. The same sampling schedule was applied to all three drying configurations to ensure comparability among treatments. Ambient temperature during the experimental period was monitored and is presented in the Results section. Moisture reduction in the mango samples was periodically evaluated throughout the drying process as an indicator of drying performance.

The three drying configurations were selected to enable a systematic comparison between passive natural-convection and forced-convection solar drying and, within the forced-convection systems, to evaluate the influence of thermal-storage location. NCD represented the simplest configuration, relying exclusively on buoyancy-driven airflow without thermal storage, whereas FCD-TSC and FCD-TSD operated under the same forced-air conditions but differed in the location of the limestone thermal-storage material. Maintaining the same mass airflow rate in both forced-convection systems allowed the effect of thermal-storage location to be evaluated under comparable airflow conditions. Limestone was selected as a sensible heat-storage material because of its ability to absorb thermal energy during periods of solar availability and subsequently release part of the stored heat as solar input decreased. Overall, the experimental configurations were designed to compare a low-complexity passive solar drying system with two forced-convection alternatives incorporating thermal storage at different locations within the drying system.

### 2.3 Sample collection and preparation

Fruits of *Mangifera indica* L. var. Keitt were used in this study. The Keitt variety was selected due to its commercial availability in the study region, high pulp yield, and suitability for dehydration processes because of its low fiber content and desirable physicochemical characteristics. The fruits were selected at their optimal physiological maturity stage based on external visual characteristics, including uniform size, characteristic peel coloration of the Keitt variety, suitable firmness, and the absence of visible defects. The samples were obtained from local markets in Chachapoyas, Amazonas region, Peru.

Although the fruits were obtained from local markets and the exact orchard origin and harvest date were not specified, efforts were made to minimize variability by selecting samples with consistent external characteristics.

Prior to processing, the fruits were washed and sanitized at the facilities of the PROCICEA project of the Universidad Nacional Toribio Rodríguez de Mendoza (UNTRM), Chachapoyas, Amazonas, Peru. The fruits were washed with potable water and disinfected in a 0.5% (w/v) sodium hypochlorite solution for 5 min, following established protocols for tropical fruits. After disinfection, the samples were thoroughly rinsed with potable water to remove residual disinfectant. This treatment was applied as a standard sanitization step to reduce surface microbial load prior to drying.

### 2.4 Mango dehydration process

After sanitization, the mangoes were manually peeled, and the pulp was cut into slices with an approximate thickness of 3 ± 0.5 mm using a stainless-steel knife to promote homogeneous heat and mass transfer during dehydration, following the procedure described by Yalta et al. (Chappa et al., 2025), with modifications. The sliced pulp obtained from multiple fruits was mixed to form a homogeneous composite sample before its allocation to the experimental treatments. From this homogenized batch, 2 kg of mango pulp were allocated to each drying system (NCD, FCD-TSC, and FCD-TSD), corresponding to a total of 6 kg of pulp used for the three drying treatments. A representative portion of the same homogenized pulp was separated before drying and used as the fresh control reference (0 min), thereby ensuring that the control and drying treatments originated from the same initial sample. The 0-min sampling point corresponded to 8:00 a.m., immediately before the mango samples were introduced into the drying systems.

The study was conducted under a completely randomized design with a 3 × 7 factorial arrangement. The first factor corresponded to the drying method (three levels: NCD, FCD-TSC, and FCD-TSD), and the second factor corresponded to drying time (seven levels: 240, 480, 1440, 1680, 1920, 2880, and 3120 min). The fresh mango sample collected before drying (0 min) was used exclusively as a non-dried reference and was not included as a level of the factorial arrangement.

The dehydration process was monitored for up to 3120 min, with measurements performed at 240, 480, 1440, 1680, 1920, 2880, and 3120 min. Consecutive daytime measurements were separated by 240 min (4 h), whereas the 480–1440 min and 1920–2880 min periods corresponded to 960-min (16-h) overnight intervals. At the end of each daily drying period, the mango samples were not removed from the drying systems but remained inside their respective drying chambers overnight. The access doors of the drying chambers remained closed during both daytime operation and the overnight period. No additional cover or covering material was placed over the dryers. Therefore, no covering or uncovering procedure was required between experimental days. Drying resumed the following day under the prevailing solar and environmental conditions. Moisture content was determined at the established sampling times to evaluate the progression of dehydration among the three drying systems. Physicochemical and bioactive analyses were performed in triplicate, whereas firmness was measured in five replicates.

### 2.5 Characterization of the physicochemical properties of dehydrated mango pulp

#### 2.5.1 pH

The pH of fresh and dehydrated mango samples was determined following the methodology described by Azumah et al. (Azumah et al., 2024), with modifications. The pH meter (LAQUA PH1300, HORIBA Scientific) was calibrated using buffer solutions at pH 4 and 7. Fresh samples were ground using a mortar, whereas dehydrated samples were pulverized using an electric mill (MKM6003, Bosch). Subsequently, 3 g of each sample were weighed into Falcon tubes, and 45 mL of distilled water were added. The mixture was homogenized using an orbital shaker (Orbi-Shaker Jr. BT302, Benchmark Scientific) for 1 h. The resulting suspensions were filtered through Whatman No. 40 filter paper, and the pH was measured using the calibrated pH meter.

#### 2.5.2 Titratable acidity

Titratable acidity of fresh and dehydrated samples was determined according to the methodology described by Suresh et al. (Suresh et al., 2025), with modifications. A suspension was prepared by homogenizing 3 g of sample with 45 mL of distilled water. A 10 mL aliquot of the resulting suspension was titrated with 0.1 N NaOH to the established endpoint. Titratable acidity was expressed as percentage of citric acid on a weight-to-weight basis (% w/w) and calculated according to Equation (1):


TA(%,w/w)=VN⁢a⁢o⁢H⁢x⁢NN⁢a⁢o⁢H⁢x⁢E⁢Wc⁢i⁢t⁢r⁢i⁢c⁢x⁢DF⁢x⁢ 1001000⁢x⁢m (1)

where: V is the volume of NaOH used in the titration (mL), N is the NaOH normality (eq L^_−_^1^^), EW is the equivalent weight of citric acid (64.04 g eq^_−_^1^^), DF is the dilution factor (45/10 = 4.5), and m is the initial mass of sample (3 g).

#### 2.5.3 Total soluble solids

Total soluble solids (TSS) were determined following the methodology described by Azumah et al. (Azumah et al., 2024), with modifications. The same homogenized sample preparation used for pH and titratable acidity determinations (3 g of sample homogenized with 45 mL of distilled water) was used for TSS analysis. The homogenate was filtered through Whatman No. 42 filter paper, and one drop of the resulting filtrate was placed on a digital refractometer (MA871, Milwaukee Instruments), previously calibrated at 25 °C. Results were expressed as degrees Brix (°Brix).

#### 2.5.4 Moisture content

To determine fruit moisture content, crucibles were first dried in an oven at 105 °C for 30 min, cooled in a desiccator, and weighed. Subsequently, 3 g of sample were placed into each crucible and dried at 105 °C until constant weight was achieved. After drying, the crucibles containing the samples were cooled in a desiccator and weighed again (Khatun et al., 2024). Moisture content was calculated using Equation (2):


Moisturecontent(%)=W1-W2W1X 100 (2)

where: *W*_1_ = weight of the fresh sample (g), *W*_2_ = weight of the dried sample (g).

#### 2.5.5 Color

Color measurements of fresh and dehydrated mango pulp were performed using a CR-400 colorimeter (Konica Minolta, Australia), recording the L*, a*, and b* parameters of the CIELab color system. The L* parameter represents lightness, ranging from 0 (black) to 100 (white); a* represents the chromatic coordinate from green (negative values) to red (positive values); and b* represents the chromatic coordinate from blue (negative values) to yellow (positive values) (Amedor et al., 2024).

The total color difference (ΔE) of dehydrated samples was calculated using the color values of fresh fruit as reference, according to Equation (3), and the Whiteness Index was calculated using Equation (4).


$$\Delta \,E=\sqrt{{\left(L*-L_{0}\right)}^{2}+{\left(a*-a_{0}\right)}^{2}+{\left(b*-b_{0}\right)}^{2}}$$ (3)

where: L*,a*,b* = color coordinates of the dehydrated mango sample. *L*_0_*, *a*_0_*, *b*_0_* = color coordinates of the fresh mango sample at 0 min, used as the reference for ΔE calculation.


$$W\,I=100-\sqrt{{\left(100-L^{*}\right)}^{2}+{\left(a^{*}\right)}^{2}+{\left(b^{*}\right)}^{2}}$$ (4)

#### 2.5.6 Firmness

Mango pulp firmness was measured following the methodology described by Segantani et al. (Segantini et al., 2017). A puncture test was performed using a texture analyzer (CT3 model, Brookfield Engineering Labs, USA) equipped with Texture Pro 1.0.19 software, a 10 kg load cell, and a cylindrical probe (TA-AVJ) fitted with a 2 mm cylindrical tip.

The test was conducted at a speed of 2 mm/s with a travel distance of 2 mm on the mango pulp samples. Results were expressed as the maximum force (Newtons, N) required to penetrate the pulp. Each determination was performed in five replicates (Dhara et al., 2023).

### 2.6 Bioactive properties of fresh and dehydrated fruits

#### 2.6.1 Preparation of the methanolic extract

The methanolic extract was prepared following the procedure described by Zhang et al. (Zhang et al., 2020), with modifications. For both fresh and dehydrated mango samples, 1 g of sample was mixed with 10 mL of 80% methanol. The extraction solvent was prepared using methanol without acidification. The samples were centrifuged at 3000 rpm for 15 min using a Pro-Analytical CR4000 benchtop centrifuge (Centurion Scientific, United Kingdom). The obtained supernatant was filtered through Whatman No. 42 filter paper, and the resulting extracts were transferred into Falcon tubes for subsequent analysis. Sample preparation was conducted under controlled conditions to minimize oxidation, including reduced exposure to light and air during extraction.

#### 2.6.2 Total phenolic content (TPC)

Total phenolic content was determined using the Folin Ciocalteu method, following the methodology described by Quispe et al. (Quispe-Sanchez et al., 2025), with modifications. Briefly, 20 μL of sample were mixed with 40 μL of 10% Folin–Ciocalteu reagent and, after 5 min, 160 μL of 7.5% (w/v) Na_2_CO_3_ were added. The mixture was kept in the dark for 60 min at room temperature. Absorbance was then measured at 765 nm using a microplate spectrophotometer (Multiskan SkyHigh, China). For quantification, a calibration curve was prepared using gallic acid as the standard (0–140 mg/L), with the regression equation *y* = 0.005×− 0.019; R^2^ = 0.991. Total phenolic content (TPC) was expressed as mg of gallic acid equivalents per gram of fresh sample (mg GAE/g).

#### 2.6.3 Antioxidant capacity by the DPPH radical scavenging assay

Antioxidant capacity was determined using the DPPH (2,2-diphenyl-1-picrylhydrazyl) radical scavenging assay following the method described by Vasconcelos (de Vasconcelos Medeiros et al., 2025), with modifications. Briefly, 20 μL of the sample extract was mixed with 130 μL of 0.1 mM DPPH solution prepared in methanol. The reaction mixture was incubated in the dark at 25 °C ± 0.5 °C for 30 min, and absorbance was measured at 525 nm using a Multiskan SkyHigh microplate spectrophotometer. The percentage of DPPH radical inhibition was first calculated according to Equation (5):


DPPHinhibition(%)=AB-ASABx 100 (5)

where A_*B*_ is the absorbance of the DPPH blank and A_*S*_ is the absorbance of the reaction mixture containing the sample extract. Subsequently, the DPPH inhibition percentage obtained for each sample was converted to Trolox equivalents using a Trolox calibration curve constructed by plotting DPPH inhibition (%) against Trolox concentration. The calibration equation *y* = 0.6719× + 44.181 (R^2^ = 0.9909), DPPH inhibition (%) represents Trolox concentration.

### 2.7 Data analysis

Statistical analysis was performed using three complementary approaches. First, a one-way analysis of variance (ANOVA) was conducted separately for each drying system (NCD, FCD-TSC, and FCD-TSD) to evaluate changes across drying times, including the fresh mango sample at 0 min as the common non-dried reference. When significant differences were detected, Tukey’s multiple comparison test was applied at *p* < 0.05. Lowercase letters were used to indicate significant differences among drying times within each drying system.

Second, a 3 × 7 factorial ANOVA was performed to evaluate the effects of drying method (DM), drying time (DT), and their interaction (DM × DT). Drying method consisted of three levels (NCD, FCD-TSC, and FCD-TSD), whereas drying time consisted of seven levels (240, 480, 1440, 1680, 1920, 2880, and 3120 min). The fresh mango sample at 0 min was not included in the factorial analysis because it had not been exposed to any drying treatment.

Third, to compare the final performance of the drying systems, a separate one-way ANOVA was conducted using the three drying methods at 3120 min. The fresh mango sample at 0 min was not included in this comparison. When significant differences were detected, Tukey’s multiple comparison test was applied at *p* < 0.05. Uppercase letters were used to indicate significant differences among drying methods at 3120 min.

Physicochemical and bioactive analyses were performed in triplicate, whereas firmness was measured in five replicates. Results are expressed as mean ± standard deviation. Prior to analysis of variance, residual normality and homogeneity of variances were evaluated using the Shapiro–Wilk and Levene tests, respectively.

Pearson correlation analysis was performed as an exploratory approach to evaluate associations among physicochemical, textural, and bioactive variables. Correlations were interpreted as indicative relationships rather than evidence of causality. All statistical analyses were conducted at a significance level of 5%.

## 3 Results and discussion

### 3.1 Environmental factors at the experimental installation site

Figure 3 presents the variation in ambient temperature recorded during the 4 days of evaluation, showing a clear diurnal pattern. During the early morning hours, temperatures were generally between approximately 12 °C and 14 °C, with the lowest values observed during Day 1. Temperature subsequently increased toward midday, with maximum values of approximately 20 °C–23 °C recorded during Days 1, 2, and 4. Day 3 showed a lower daytime temperature profile, reaching a maximum of approximately 16 °C–17 °C. Thereafter, temperatures decreased progressively during the afternoon and nighttime periods, approaching approximately 13 °C–15 °C by the end of the daily cycle. These results demonstrate the temporal variability in ambient temperature experienced by the solar drying systems during the experimental period.

**FIGURE 3:**
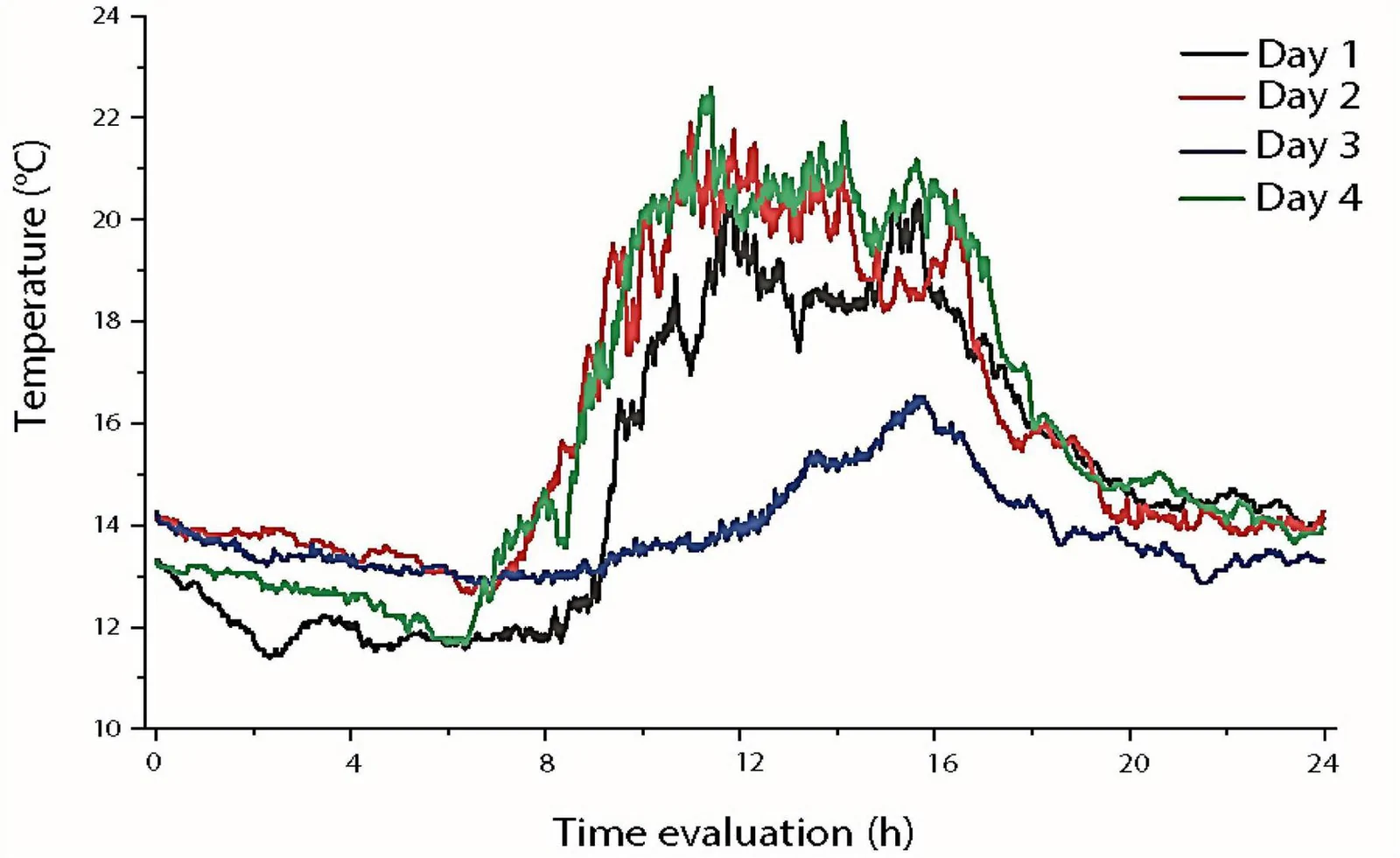
Ambient temperature variation during the 4-days experimental drying period.

### 3.2 Physicochemical properties evaluated in dehydrated mango pulp

#### 3.2.1 pH, titratable acidity, total soluble solids and moisture

Table 1 presents the changes in pH, titratable acidity, total soluble solids (TSS), and moisture content of mango pulp during solar drying. The fresh mango used as the non-dried reference (0 min; 8:00 a.m.) had a pH of 4.22 ± 0.04, titratable acidity of 0.66% ± 0.03% citric acid, TSS of 9.00 ± 0.01 °Brix, and moisture content of 86.81% ± 0.23%. The factorial analysis showed significant effects of drying method, drying time, and their interaction on pH, titratable acidity, and moisture content (*p* < 0.001 for all effects). TSS was also significantly affected by drying method (*p* < 0.05), drying time (*p* < 0.001), and the drying method × drying time interaction (*p* < 0.001).

**TABLE 1:** Physicochemical properties of mango pulp during drying using different solar drying systems.

Drying method	Drying time (min)	pH	Titratable acidity (% citric acid)	TSS (° Brix)	Moisture (%)
NCD	0 (control)	4.22 ± 0.04^a^	0.66 ± 0.03^g^	9.00 ± 0.01^g^	86.81 ± 0.23^a^
	240	3.59 ± 0.02^f^	0.62 ± 0.03^g^	13.25 ± 0.43^f^	72.09 ± 0.81^b^
	480	3.92 ± 0.02^d^	0.85 ± 0.04^f^	16.63 ± 0.22^e^	70.56 ± 0.72^b^
	1440	4.03 ± 0.01^c^	0.97 ± 0.02^e^	18.00 ± 0.87^e^	65.25 ± 1.69^c^
	1680	4.12 ± 0.01^b^	1.11 ± 0.03^d^	22.62 ± 0.21^d^	45.02 ± 0.90^d^
	1920	3.95 ± 0.04^d^	1.42 ± 0.02^c^	31.50 ± 1.50^c^	39.33 ± 0.38^e^
	2880	3.90 ± 0.01^de^	1.62 ± 0.03^b^	35.25 ± 0.75^b^	25.76 ± 0.85^f^
	3120	3.85 ± 0.01^e, A^	1.77 ± 0.03^a, B^	43.00 ± 0.87^a, AB^	21.67 ± 0.83^g, A^
FCD-TSD	0 (control)	4.22 ± 0.04^a^	0.66 ± 0.03^g^	9.00 ± 0.01^g^	86.81 ± 0.23^a^
	240	3.76 ± 0.01^e^	1.07 ± 0.04^f^	8.94 ± 0.11^g^	65.16 ± 0.28^b^
	480	3.78 ± 0.01^e^	1.19 ± 0.04^e^	12.00 ± 0.00^f^	64.08 ± 0.12^b^
	1440	3.94 ± 0.01^bc^	1.39 ± 0.01^d^	16.37 ± 0.22^e^	54.30 ± 0.93^c^
	1680	3.97 ± 0.01^b^	1.96 ± 0.04^c^	25.75 ± 0.43^d^	23.39 ± 1.13^d^
	1920	3.91 ± 0.02^cd^	2.22 ± 0.04^b^	37.13 ± 0.38^c^	18.50 ± 1.04^e^
	2880	3.88 ± 0.01^d^	2.31 ± 0.05^b^	39.57 ± 0.68^b^	17.35 ± 0.12^e^
	3120	3.86 ± 0.01^d, A^	2.44 ± 0.02^a, A^	45.00 ± 0.87^a, A^	12.24 ± 0.36^f, C^
FCD-TSC	0 (control)	4.22 ± 0.04^a^	0.66 ± 0.03^g^	9.00 ± 0.01^g^	86.81 ± 0.23^a^
	240	3.43 ± 0.01^f^	1.09 ± 0.01^f^	13.25 ± 0.43^f^	75.91 ± 1.42^b^
	480	3.58 ± 0.01^e^	1.20 ± 0.04^e^	16.37 ± 0.22^e^	67.39 ± 0.57^c^
	1440	3.72 ± 0.03^d^	1.27 ± 0.01^e^	17.63 ± 0.38^e^	60.90 ± 0.71^d^
	1680	3.90 ± 0.02^b^	1.72 ± 0.01^d^	24.00 ± 1.30^d^	44.60 ± 0.94^e^
	1920	3.83 ± 0.02^c^	1.89 ± 0.03^c^	34.12 ± 0.65^c^	22.98 ± 0.63^f^
	2880	3.80 ± 0.03^c^	2.23 ± 0.02^b^	36.67 ± 0.76^b^	20.07 ± 0.73^g^
	3120	3.72 ± 0.01^d, B^	2.51 ± 0.05^a, A^	40.87 ± 0.99^a, B^	18.70 ± 0.73^g, B^
Drying method (DM)	***	***	*	***	***
Drying time (DT)	***	***	***	***	***
DM × DT	***	***	***	***	***

Values are presented as mean ± standard deviation (*n* = 3). Each drying method was analyzed separately, including the fresh mango control (0 min). Within each drying method, different lowercase letters in the same column indicate significant differences among drying times according to Tukey’s multiple comparison test (*p* < 0.05). Means sharing at least one lowercase letter are not significantly different. Different uppercase letters associated with values at 3120 min indicate significant differences among drying methods according to Tukey’s multiple comparison test (*p* < 0.05); means sharing at least one uppercase letter are not significantly different. The effects of drying method (DM), drying time (DT), and their interaction (DM × DT), shown at the bottom of the table, were evaluated using a 3 × 7 factorial ANOVA. The fresh control (0 min) was excluded from the factorial analysis because it had not been exposed to any drying treatment. NCD, natural convection dryer without thermal storage; FCD-TSC, forced convection dryer with thermal storage in the solar collector; FCD-TSD, forced convection dryer with thermal storage in the drying chamber; TSS, total soluble solids; ns, not significant;

*p < 0.05;

**p < 0.01;

***p < 0.001.

For NCD, all evaluated physicochemical parameters showed significant changes during drying (*p* < 0.05). The pH initially decreased from 4.22 ± 0.04 in the fresh control to 3.59 ± 0.02 at 240 min, subsequently increased to 4.12 ± 0.01 at 1680 min, and reached 3.85 ± 0.01 at 3120 min. Titratable acidity increased from 0.66% ± 0.03% in the fresh control to 1.77% ± 0.03% at 3120 min, while TSS increased from 9.00 ± 0.01 to 43.00 ± 0.87 °Brix. In parallel, moisture content decreased progressively from 86.81% ± 0.23% to 21.67% ± 0.83%. These changes indicate progressive water removal accompanied by concentration of soluble solids and titratable acids during natural-convection drying.

For FCD-TSC, significant temporal changes were also observed in pH, titratable acidity, TSS, and moisture content (*p* < 0.05). The pH decreased from 4.22 ± 0.04 in the fresh control to 3.43 ± 0.01 at 240 min, subsequently increased to 3.90 ± 0.02 at 1680 min, and reached 3.72 ± 0.01 at 3120 min. Titratable acidity increased from 0.66% ± 0.03% to 2.51% ± 0.05%, while TSS increased from 9.00 ± 0.01 to 40.87 ± 0.99 °Brix. Moisture content decreased from 86.81% ± 0.23% in the fresh control to 18.70% ± 0.73% at the end of drying. These changes are consistent with progressive water removal and the associated concentration of soluble constituents during fruit dehydration (Yanclo et al., 2024).

For FCD-TSD, all four physicochemical parameters showed significant changes across drying time (*p* < 0.05). The pH decreased from 4.22 ± 0.04 in the fresh control to 3.76 ± 0.01 at 240 min and subsequently varied throughout the drying period, reaching 3.86 ± 0.01 at 3120 min. Titratable acidity increased from 0.66% ± 0.03% to 2.44% ± 0.02%, whereas TSS increased from 9.00 ± 0.01 to 45.00 ± 0.87 °Brix. Moisture content decreased markedly from 86.81% ± 0.23% in the fresh control to 12.24% ± 0.36% at 3120 min. Therefore, FCD-TSD showed progressive moisture removal accompanied by increases in titratable acidity and soluble solids during dehydration.

The separate comparison among drying methods at 3120 min revealed significant differences in final pH. NCD (3.85 ± 0.01; A) and FCD-TSD (3.86 ± 0.01; A) did not differ significantly from each other, whereas FCD-TSC (3.72 ± 0.01; B) showed a significantly lower pH (*p* < 0.05). These values remained within the acidic range commonly reported for ripe mangoes (Santo et al., 2008, Zongo et al., 2022). Changes in pH during dehydration may be associated with the concentration and transformation of organic acids and other soluble constituents as water is removed from the fruit matrix (Dereje and Abera, 2020). Acidic conditions are also relevant to processed fruit stability because they can restrict the growth of several microorganisms (Etienne et al., 2013).

Final titratable acidity also differed among drying systems. FCD-TSD (2.44% ± 0.02%; A) and FCD-TSC (2.51% ± 0.05%; A) did not differ significantly from each other, whereas NCD (1.77% ± 0.03%; B) showed significantly lower titratable acidity (*p* < 0.05). The increase in titratable acidity relative to fresh mango may be primarily associated with the concentration of organic acids as water is removed during dehydration (Yanclo et al., 2024, Yanclo et al., 2024). The different behavior of pH and titratable acidity is expected because titratable acidity represents the overall acid content, whereas pH reflects hydrogen ion activity and is additionally influenced by buffering capacity and acid dissociation equilibria.

At 3120 min, TSS was highest in FCD-TSD (45.00 ± 0.87 °Brix; A), followed by NCD (43.00 ± 0.87 °Brix; AB) and FCD-TSC (40.87 ± 0.99 °Brix; B). FCD-TSD and FCD-TSC differed significantly (*p* < 0.05), whereas NCD, which belonged to the intermediate homogeneous group AB, did not differ significantly from either FCD-TSD or FCD-TSC. The increase in TSS relative to the fresh mango reference (9.00 ± 0.01 °Brix) is consistent with the concentration of soluble constituents resulting from progressive water removal during dehydration (Mishra et al., 2021).

Final moisture content differed significantly among the three drying systems (*p* < 0.05). FCD-TSD showed the lowest moisture content (12.24% ± 0.36%; C), followed by FCD-TSC (18.70% ± 0.73%; B), whereas NCD presented the highest final moisture content (21.67% ± 0.83%; A). Thus, under the evaluated experimental conditions and at the common final drying time of 3120 min, FCD-TSD achieved significantly greater moisture reduction than FCD-TSC and NCD. Nevertheless, these results describe moisture removal at a fixed drying time and should not be interpreted as evidence of microbiological stability or shelf life. Because water activity (Aw) and microbiological parameters were not determined, the implications of the final moisture levels for product stability and storage behavior cannot be directly established.

Overall, the final-time comparison demonstrated that the effect of dryer configuration depended on the physicochemical attribute considered. FCD-TSC showed the lowest final pH, FCD-TSD and FCD-TSC showed higher titratable acidity than NCD, FCD-TSD showed higher TSS than FCD-TSC with NCD occupying an intermediate position, and FCD-TSD achieved the lowest final moisture content. Therefore, the results do not identify a single drying configuration as superior for all physicochemical attributes, although FCD-TSD showed the greatest moisture removal at the common final drying time.

#### 3.2.2 Color changes

Color parameters varied according to drying method and drying time (Table 2). The factorial analysis showed that drying method and the drying method × drying time interaction significantly affected all color parameters (*p* < 0.001). Drying time also significantly affected L*, a*, WI, and ΔE (*p* < 0.001), as well as b* (*p* < 0.01).

**TABLE 2:** Color parameters of dehydrated mango pulp evaluated under different solar drying methods and drying times.

Drying method	Drying time (min)	L*	a*	b*	WI	ΔE
NCD	0 (control)	58.28 ± 0.02^abc^	2.87 ± 0.28^d^	43.38 ± 0.04^ab^	39.75 ± 0.01^ab^	0.00 ± 0.00^c^
	240	63.37 ± 1.71^a^	3.51 ± 0.99^cd^	50.77 ± 3.30^a^	37.24 ± 1.70^b^	9.04 ± 3.72^abc^
	480	62.54 ± 2.89^ab^	4.27 ± 1.00^b, d^	48.57 ± 3.13^a^	38.42 ± 1.09^ab^	7.09 ± 3.80^bc^
	1440	60.91 ± 1.44^a, c^	5.63 ± 0.28^a, d^	48.42 ± 2.77^a^	37.47 ± 1.24^b^	6.44 ± 2.77^bc^
	1680	54.62 ± 3.82^a, d^	6.35 ± 0.75^a, c^	43.72 ± 2.59^ab^	36.56 ± 1.46^b^	6.21 ± 1.57^bc^
	1920	46.73 ± 2.19^de^	8.92 ± 1.86^a^	35.13 ± 2.87^bc^	35.49 ± 0.64^b^	15.51 ± 3.57^ab^
	2880	45.65 ± 4.09^e^	7.54 ± 2.17^ab^	29.69 ± 2.24^c^	37.58 ± 4.64^b^	19.62 ± 1.49^a^
	3120	53.35 ± 3.88^c, e, B^	7.97 ± 0.16^a, A^	25.53 ± 9.97^c, A^	45.67 ± 4.89^a, B^	19.69 ± 9.28^a, A^
FCD-TSD	0 (control)	58.28 ± 0.02^e^	2.87 ± 0.28^a^	43.38 ± 0.04^b^	39.75 ± 0.01^bc^	0.00 ± 0.00^d^
	240	78.65 ± 0.73^a^	0.76 ± 0.09^bc^	59.69 ± 2.05^a^	36.59 ± 1.69^c^	26.2 ± 1.86^a^
	480	76.63 ± 1.69^ab^	−1.15 ± 0.12^de^	48.86 ± 2.03^ab^	45.79 ± 1.11^abc^	19.62 ± 2.11^a, c^
	1440	72.36 ± 2.63^cd^	0.42 ± 0.36^cd^	37.72 ± 14.06^b^	52.65 ± 11.04^a^	18.42 ± 7.09^a, c^
	1680	78.38 ± 0.67^a^	−1.49 ± 0.96^e^	44.33 ± 1.96^b^	50.63 ± 1.45^ab^	20.67 ± 0.50^ab^
	1920	70.29 ± 0.95^d^	3.03 ± 1.24^a^	42.27 ± 2.13^b^	48.21 ± 1.25^ab^	12.24 ± 0.60^c^
	2880	74.66 ± 0.77^a, c^	3.14 ± 0.21^a^	45.40 ± 1.12^ab^	47.91 ± 1.30^abc^	16.54 ± 0.69^a, c^
	3120	77.20 ± 0.38^ab, A^	2.34 ± 0.46^ab, B^	40.16 ± 1.15^b, A^	53.76 ± 1.10^a, A^	19.22 ± 0.49^a, c, A^
FCD-TSC	0 (control)	58.28 ± 0.02^bc^	2.87 ± 0.28^c^	43.38 ± 0.04^a, c^	39.75 ± 0.01^c, e^	0.00 ± 0.00^c^
	240	65.92 ± 1.31^a^	3.06 ± 0.53^c^	50.28 ± 2.09^a^	39.15 ± 1.04^de^	10.33 ± 2.35^b^
	480	65.75 ± 0.81^a^	3.62 ± 0.25^c^	49.74 ± 4.79^a^	39.43 ± 3.49^de^	10.25 ± 3.39^b^
	1440	64.92 ± 0.88^a^	2.62 ± 1.00^c^	48.35 ± 2.96^ab^	40.17 ± 1.99^cd^	8.52 ± 2.24^bc^
	1680	49.76 ± 0.15^d^	10.44 ± 1.29^a^	39.28 ± 0.62^bcd^	35.37 ± 0.54^e^	12.15 ± 0.66^ab^
	1920	60.65 ± 0.54^ab^	8.67 ± 1.09^ab^	31.62 ± 2.37^de^	48.75 ± 1.33^a^	13.44 ± 1.64^ab^
	2880	52.30 ± 2.32^cd^	6.65 ± 0.29^b^	23.95 ± 3.12^e^	46.13 ± 0.85^ab^	20.71 ± 3.58^a^
	3120	56.73 ± 5.20^bc, B^	7.42 ± 1.08^b, A^	33.80 ± 6.52^cd, A^	44.17 ± 0.92^bc, B^	11.75 ± 5.97^b, A^
Drying method (DM)	***	***	***	***	***	***
Drying time (DT)	***	***	**	***	***	***
DM × DT	***	***	***	***	***	***

Values are presented as mean ± standard deviation (*n* = 3). Each drying method was analyzed separately, including the fresh mango control (0 min). Within each drying method, different lowercase letters in the same column indicate significant differences among drying times according to Tukey’s multiple comparison test (*p* < 0.05). Means sharing at least one letter are not significantly different, whereas means with no letters in common are significantly different. Combined letters indicate membership in multiple homogeneous groups; for example, “ab” indicates membership in groups a and b, whereas “a–c” is used as compact notation for “abc” (groups a, b, and c). Different uppercase letters at 3120 min indicate significant differences among drying methods according to Tukey’s multiple comparison test (*p* < 0.05). The effects of drying method (DM), drying time (DT), and their interaction (DM × DT), shown at the bottom of the table, were evaluated using a 3 × 7 factorial ANOVA; the fresh control (0 min) was excluded from the factorial analysis because it had not been exposed to any drying treatment. NCD, natural convection dryer without thermal storage; FCD-TSC, forced convection dryer with thermal storage in the solar collector; FCD-TSD, forced convection dryer with thermal storage in the drying chamber; WI, whiteness index; ΔE, total color difference; ns, not significant;

*p < 0.05;

**p < 0.01;

***p < 0.001.

The separate within-dryer analysis showed different temporal responses among drying systems. For NCD, L* decreased significantly during drying (*p* < 0.05), indicating a reduction in lightness over time. In contrast, L* did not change significantly in FCD-TSD (*p* > 0.05), whereas significant temporal changes were observed in FCD-TSC (*p* < 0.05). These differences in lightness may be associated with changes in moisture content, which influence color reflectance (García-Valladares et al., 2024), as well as with prolonged exposure to drying conditions that may promote browning reactions and color modifications (Lucho-Gómez et al., 2026).

The a* coordinate showed significant temporal changes in NCD and FCD-TSC (*p* < 0.05), whereas no significant change was detected within FCD-TSD (*p* > 0.05). The numerical increase observed particularly in NCD and FCD-TSC indicates a shift toward more pronounced red-orange tones. This behavior may be associated with browning reactions (Bulantekin and Kuşçu, 2024, Fijalkowska et al., 2016), and the concentration or transformation of pigments as water is removed. The b* coordinate remained positive in all treatments, confirming the predominance of yellow tones characteristic of mango pulp. Significant temporal changes in b* were observed in all three drying systems (*p* < 0.05). The numerical decrease observed at prolonged drying times, particularly in NCD and FCD-TSC, may be associated with changes in carotenoid pigments and browning reactions during drying (Nowacka et al., 2021).

The whiteness index (WI) showed a significant temporal change within FCD-TSD (*p* < 0.05), whereas the changes observed within NCD and FCD-TSC were not statistically significant (*p* > 0.05). In contrast, ΔE did not show significant temporal changes within any of the three drying systems (*p* > 0.05). Nevertheless, the magnitude of ΔE indicated perceptible chromatic differences relative to the fresh mango reference. ΔE values greater than 3 are generally considered noticeable, whereas values between 1.5 and 3 represent detectable color differences (Patras, 2019). Therefore, the observed ΔE values indicate perceptible color modifications during dehydration, although the temporal differences within each drying system were not statistically significant.

The separate comparison at 3120 min showed that FCD-TSD had a significantly higher L* and a significantly lower a* than the other drying systems (*p* < 0.05), indicating greater final lightness and a lower shift toward red tones. In contrast, final WI and ΔE did not differ significantly among the drying systems (*p* > 0.05). Therefore, the statistical evidence supports differences among drying systems for specific final color coordinates, particularly L* and a*, but does not demonstrate an overall superiority of FCD-TSD for all color parameters. The greater darkening numerically observed in NCD during prolonged drying is consistent with color changes previously reported in dehydrated fruits (Azhar and Yusof, 2026).

#### 3.2.3 Firmness

Firmness is defined as the force required to compress a food to a given distance and is associated with the product’s resistance to deformation (Biswas et al., 2025). Firmness increased with drying time. Textural changes during drying are associated with complex structural modifications in plant tissue, including cell wall degradation, pectin solubilization, and loss of turgor pressure. These processes lead to changes in mechanical properties such as firmness and elasticity, which are influenced by both moisture content and drying conditions.

Figure 4 shows that firmness increased progressively with drying time in all treatments, being minimal in the control and maximal at prolonged times (2880–3120 min). The increase was more pronounced in NCD and FCD-TSD, whereas FCD-TSC presented relatively lower firmness values at the end of the process. In general, the results indicate that both drying method and time influenced product firmness, with greater hardening associated with extended drying periods. It is important to note that differences in final moisture content among treatments may influence the comparability of quality parameters. Therefore, the observed variations should be interpreted considering the drying dynamics and specific conditions of each system rather than as strictly equivalent final states.

**FIGURE 4:**
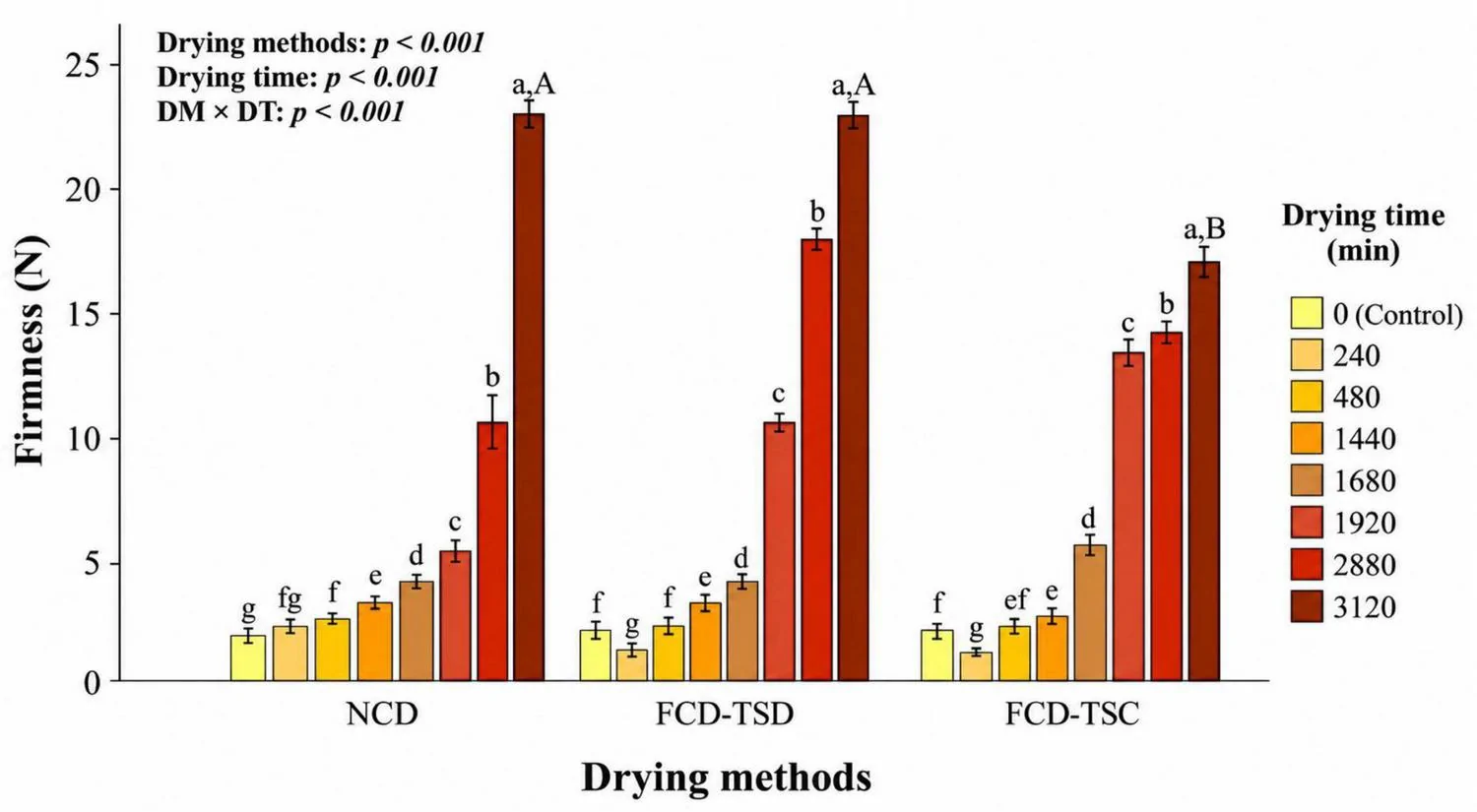
Changes in mango pulp firmness **(N)** as a function of drying time (0–3120 min) and drying methods (NCD, FCD-TSD, and FCD-TSC). Each drying method was analyzed separately, including the fresh mango control (0 min). Different lowercase letters indicate significant differences among drying times within each drying method, whereas different uppercase letters at 3120 min indicate significant differences among drying methods according to Tukey’s multiple comparison test (*p <* 0.05). DM, drying method; DT, drying time; DM × DT, interaction between drying method and drying time.

Drying temperature is a determining factor in the textural properties of dehydrated fruits; high temperatures tend to increase firmness and brittleness, whereas lower temperatures favor greater cohesion and gumminess (Aggarwal et al., 2022). The increase in firmness is due to moisture removal and the concentration of other components during the dehydration process (Pashazadeh et al., 2021). Textural changes during drying are associated with complex structural modifications in plant tissue, including cell wall degradation, pectin solubilization, and loss of turgor pressure. These processes lead to changes in mechanical properties such as firmness and elasticity, which are influenced by both moisture content and drying conditions Chong et al. (Chong et al., 2008), associated the increase in firmness observed during dehydration with the depolymerization of cell wall components, such as pectin.

#### 3.2.4 Total phenolic content (TPC)

Polyphenolic compounds are non-nutritive secondary metabolites present in fruits and are recognized for their contribution to human health due to their diverse bioactive properties. Figure 5 shows that the measured total phenolic content of dehydrated mango pulp increased progressively with drying time and depended on the drying method. Fresh mango (control) presented the lowest phenolic content (0.77 ± 0.01 mg GAE/g), whereas higher values were recorded at prolonged drying times. At 3120 min, FCD-TSD showed the highest TPC (27.89 ± 0.46 mg GAE/g), followed by FCD-TSC (25.16 ± 0.24 mg GAE/g) and NCD (22.07 ± 0.25 mg GAE/g). Thus, under the evaluated conditions, FCD-TSD resulted in the highest measured TPC at the end of the drying period.

**FIGURE 5:**
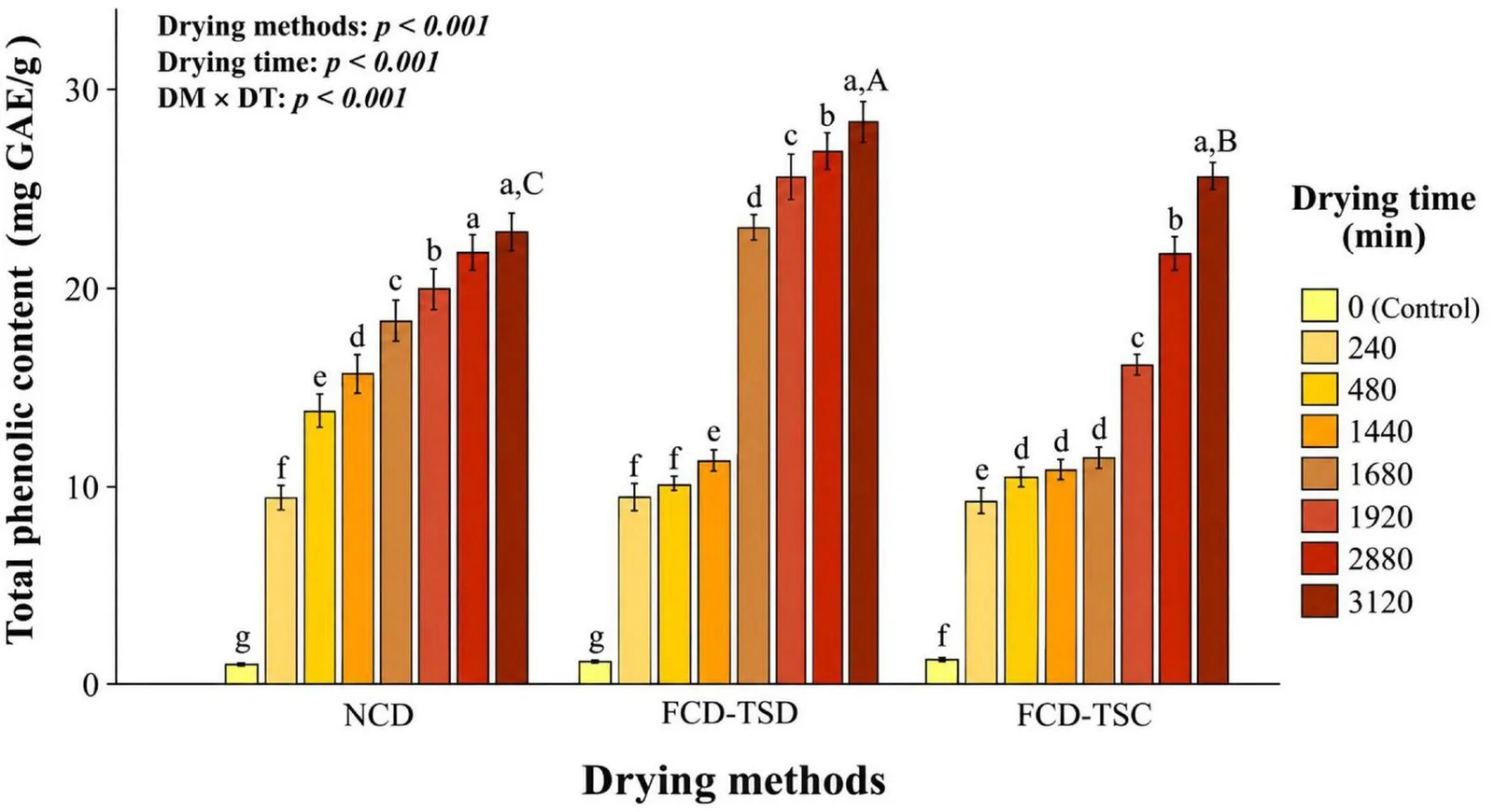
Changes in total phenolic content of mango pulp as a function of drying time (0–3120 min) and drying methods (NCD, FCD-TSD, and FCD-TSC). Each drying method was analyzed separately, including the fresh mango control (0 min). Different lowercase letters indicate significant differences among drying times within each drying method, whereas different uppercase letters at 3120 min indicate significant differences among drying methods according to Tukey’s multiple comparison test (*p* < 0.05). DM, drying method; DT, drying time; DM × DT, interaction between drying method and drying time.

The apparent increase in TPC during dehydration should be interpreted with caution and should not be considered direct evidence of increased retention or net formation of phenolic compounds. Progressive moisture loss concentrates the solid fraction of the sample and may consequently increase the measured phenolic content per unit mass (Yanclo et al., 2024, Mugodo and Workneh, 2021). In addition, drying induced structural changes in the fruit matrix may enhance the extractability of bound phenolic compounds, thereby contributing to higher measured TPC values. Conversely, thermal exposure during prolonged drying may promote degradation of thermolabile phenolic compounds and the formation of non-enzymatic browning products with reducing activity. Therefore, the observed TPC values likely reflect the combined effects of moisture concentration, changes in phenolic extractability, and thermal degradation rather than an unequivocal increase in the absolute number of phenolic compounds. Drying conditions may consequently influence the balance between these mechanisms and the measured phenolic content of dehydrated mango (Zeng et al., 2024).

#### 3.2.5 Antioxidant capacity

Figure 6 shows that antioxidant capacity increased with drying time in all evaluated methods. The control presented the lowest value (3.88 ± 0.07 μmol TE/g), whereas the drying treatments showed progressive increases, especially at prolonged times. The highest value was obtained in NCD at 3120 min, reaching 9.43 ± 0.07 μmol TE/g, followed by FCD-TSD at 3120 min with 8.53 ± 0.01 μmol TE/g and FCD-TSC at 3120 min with 8.49 ± 0.03 μmol TE/g. In general, the results indicate that extended drying times favored the increase in antioxidant capacity, with NCD being the method that achieved the highest values at the end of the process.

**FIGURE 6:**
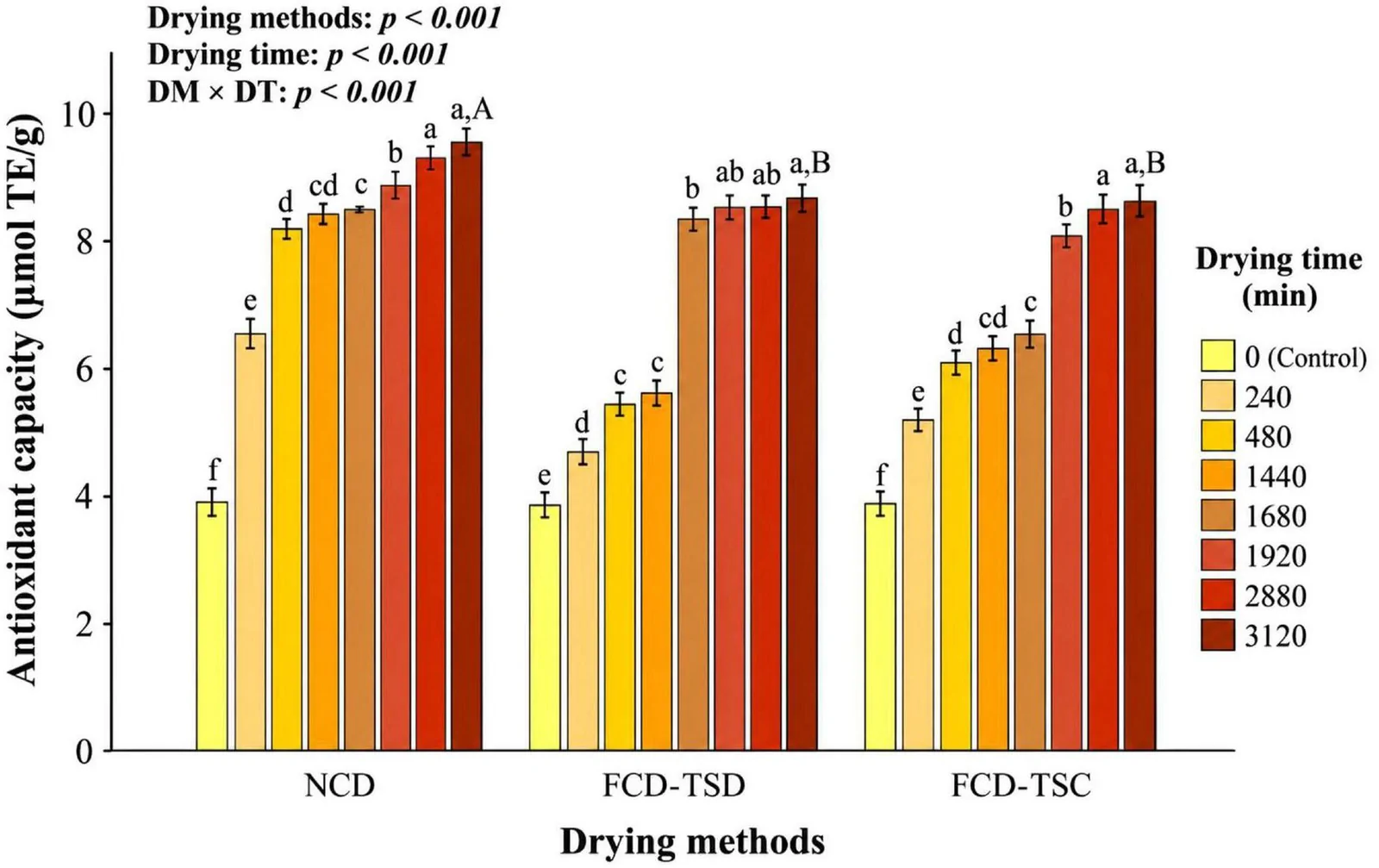
Changes in antioxidant capacity of mango pulp as a function of drying time (0–3120 min) and drying methods (NCD, FCD-TSD, and FCD-TSC). Each drying method was analyzed separately, including the fresh mango control (0 min). Different lowercase letters indicate significant differences among drying times within each drying method, whereas different uppercase letters at 3120 min indicate significant differences among drying methods according to Tukey’s multiple comparison test (*p* < 0.05). DM, drying method; DT, drying time; DM × DT, interaction between drying method and drying time.

Hot air drying reduces the water content of mango, which promotes the concentration of antioxidant compounds and results in an increase in antioxidant capacity when expressed on a dry basis (Miraei Ashtiani et al., 2022).

The observed changes in total phenolic content and antioxidant capacity should not be attributed exclusively to dry matter concentration. During drying, structural disruption of the mango matrix may enhance the extractability of bound phenolic compounds, while thermal exposure may simultaneously promote degradation of thermolabile compounds or the formation of non-enzymatic browning products with antioxidant capacity. Therefore, the final bioactive response reflects a balance between concentration effects, compound release, degradation, and possible formation of antioxidant-active reaction products.

#### 3.2.6 Pearson correlation

Pearson correlation analysis revealed a consistent pattern across the three evaluated drying configurations: natural convection drying without thermal storage (NCD), forced convection drying with thermal storage in the solar collector (FCD-TSC), and forced convection drying with thermal storage in the drying chamber (FCD-TSD) as shown in Figure 7. Moisture acted as a dominant structural variable of the drying process. In all systems, moisture exhibited strong to nearly perfect negative correlations with titratable acidity (TTA), total soluble solids (TSS), firmness, total phenolic content (TPC), and antioxidant capacity measured by DPPH (—r| ≥ 0.77; *p* < 0.01). These relationships indicate that the progression of drying and water removal was closely associated with changes in physicochemical, textural, and bioactive variables. However, these associations should be interpreted cautiously because several variables changed simultaneously with drying time and decreasing moisture content.

**FIGURE 7:**
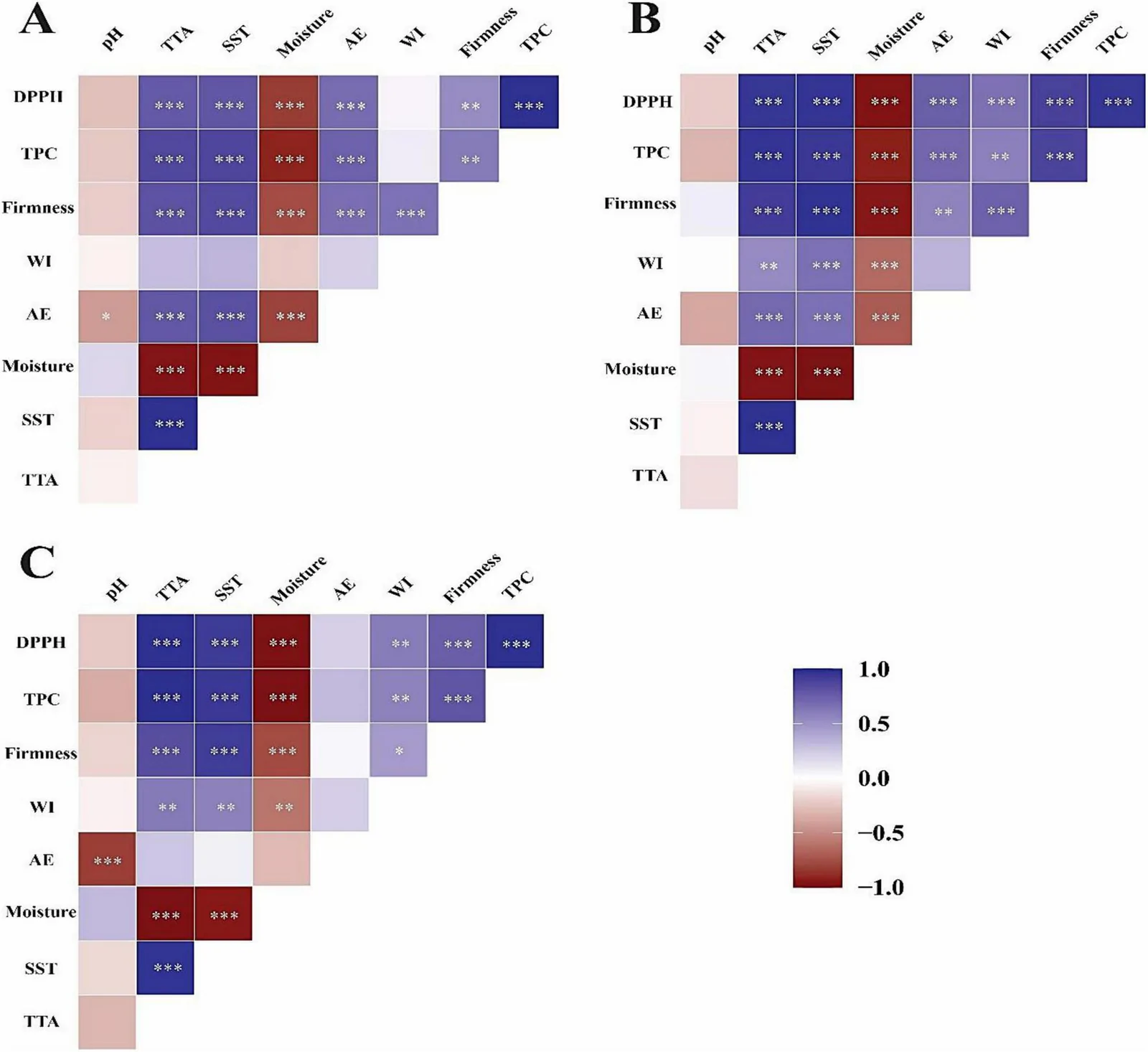
Pearson correlation heatmaps showing the relationships among key physicochemical, textural, and bioactive parameters of dehydrated mango under the different solar drying configurations: **(A)** natural convection dryer without thermal storage (NCD), **(B)** forced convection dryer with thermal storage in the solar collector (FCD-TSC), and **(C)** forced convection dryer with thermal storage in the drying chamber (FCD-TSD). Correlations are presented as exploratory associations and should not be interpreted as evidence of causal relationships. The symbols indicate the statistical significance levels of the Pearson correlations: *p < 0.05, **p < 0.01, and ***p < 0.001.

Titratable acidity and TSS showed strong positive correlations with each other (r ≈ 0.97–0.98; *p* < 0.001) and strong negative associations with moisture (r ≈ −0.94 to −0.99; *p* < 0.001) across the three configurations, indicating their role as indicators of dehydration progress. Similar trends have been reported in osmoconvective dehydration processes of banana peel, where water loss led to increases in soluble solids and total solids (Silva et al., 2020). Both variables were positively correlated with firmness, TPC, and DPPH, suggesting that structural changes and apparent enrichment in bioactive compounds occurred simultaneously during drying.

Firmness was negatively correlated with moisture and positively associated with TTA, TSS, TPC, and DPPH, reflecting the progressive formation of denser and more com-pact structures as water content decreased (Dadhaneeya et al., 2023, García-Moreira et al., 2023), Water loss is known to increase soluble solids and, in some cases, titratable acidity (Kolla et al., 2021, El-Beltagi et al., 2023), while appropriate drying conditions may preserve phenolic compounds and antioxidant capacity. The positive association observed between TPC and DPPH suggests that phenolic compounds may contribute substantially to antioxidant capacity, as commonly reported in fruits (Gu et al., 2019). However, these associations should be interpreted with caution, as they may also reflect concentration effects and changes in extractability during drying.

In contrast, pH did not show significant correlations with most evaluated variables (*p* > 0.05), indicating a more independent behavior. Its only relevant association was observed with total color change (ΔE), particularly in FCD-TSD (*r* = −0.82; *p* < 0.001). Chromatic parameters (ΔE and WI) showed variable relationships depending on the drying system, reflecting the influence of temperature and irradiance on color development (García-Moreira et al., 2025, García-Moreira et al., 2025).

Overall, the results suggest that solar drying dynamics were characterized by an interconnected pattern involving moisture, concentration-related variables (TTA and TSS), structural properties (firmness), and bioactive indicators (TPC and DPPH), with stronger associations under forced-convection conditions. However, these correlations should be interpreted as exploratory associations rather than evidence of direct causal relationships. Because several physicochemical and bioactive variables changed concurrently with drying time and progressive moisture removal, part of the observed correlation structure may reflect their common response to water loss. In particular, increases in variables expressed per unit mass may partly result from concentration effects as moisture decreases. Therefore, strong correlations among physicochemical and bioactive parameters do not necessarily indicate direct mechanistic relationships between them.

It is important to note that the correlations observed in this study, particularly those involving moisture content, may be influenced by the intrinsic interdependence of water-related variables during the drying process. Therefore, these relationships should be interpreted as indicative associations rather than direct causal effects. In addition, the strong correlations between moisture and other parameters may partly reflect concentration effects associated with water loss, which should be considered when interpreting the results. Consequently, Pearson correlation analysis in this context serves as an exploratory tool rather than definitive evidence of mechanistic relationships.

Although the analytical methods employed in this study provide a comprehensive evaluation of physicochemical and functional properties, they offer a global characterization rather than a compound-specific analysis. Although the interpretation of bioactive compound changes was carefully limited to concentration effects associated with dehydration, the absence of complete standardization of all measurements on a common dry weight basis represents a methodological limitation for direct comparison between fresh and dried samples. Therefore, the reported variations in total phenolic content and antioxidant capacity should be interpreted primarily as comparative indicators of dehydration-related changes under the evaluated drying conditions rather than as absolute increases in bioactive compounds.

An additional limitation of this study is the absence of detailed solar radiation measurements and thermal balance analysis of the drying systems. Although the drying process was successfully completed under the evaluated environmental conditions, future studies should incorporate energetic performance assessments and advanced analytical techniques such as high performance liquid chromatography (HPLC) to better understand heat transfer dynamics, molecular changes, and system efficiency under high cloudiness conditions.

Additionally, water activity (aw) was not determined in this study, although it is an important parameter associated with microbial stability and shelf life in dehydrated foods. Future research should include aw measurements to provide a more comprehensive evaluation of product stability and storage quality.

The thermophysical properties of the limestone used for thermal storage, including mass and specific heat capacity, were also not experimentally determined. Future studies should incorporate detailed characterization of the storage material and thermal performance analysis of the drying systems.

Furthermore, texture evaluation was limited to firmness measurements. Additional texture profile parameters, including cohesiveness, springiness, gumminess, and chewiness, should be incorporated in future studies to provide a more comprehensive characterization of the mechanical properties of dehydrated mango.

Antioxidant capacity was evaluated only using the DPPH assay, which provides a general estimate of radical scavenging activity but does not fully represent the complexity of antioxidant reactions in the mango matrix. Future studies should incorporate complementary methods such as ABTS, FRAP, or ORAC to provide a more comprehensive assessment of antioxidant capacity.

## 4 Conclusion

This study demonstrated that solar dryer configuration and drying time significantly influenced the physicochemical, textural, and bioactive characteristics of mango under the climatic conditions of the Peruvian Amazon. At the common final drying time of 3120 min, FCD-TSD achieved the lowest moisture content (12.24% ± 0.36%), followed by FCD-TSC (18.70% ± 0.73%) and NCD (21.67% ± 0.83%), with significant differences among the three systems. However, the relative performance of the dryers depended on the quality attribute considered. FCD-TSC showed the lowest final pH, FCD-TSD and FCD-TSC showed higher titratable acidity than NCD, and FCD-TSD showed higher TSS than FCD-TSC, while NCD occupied an intermediate position. Therefore, no single drying configuration was superior for all evaluated quality attributes.

The increases observed in measured total phenolic content and antioxidant capacity during drying should not be interpreted as unequivocal evidence of greater retention or formation of bioactive compounds. These responses likely reflect the combined effects of moisture concentration, changes in compound extractability, and thermal exposure. From a practical perspective, the results demonstrate the potential of forced-convection solar drying combined with sensible thermal storage as an alternative for mango dehydration under variable solar conditions. However, confirmation of microbiological stability and shelf-life requires additional evaluation of water activity, microbiological quality, and storage behavior.

Future research should incorporate compound-specific analytical techniques, dry-weight-based expression of bioactive compounds, water activity and microbiological analyses, and process optimization under different environmental conditions. Energy and economic assessments are also required before extrapolating the performance of these prototype systems to larger-scale applications.

## Data Availability

The original contributions presented in this study are included in the article/Supplementary material, further inquiries can be directed to the corresponding authors.
